# Canada and the Convention on International Trade in Endangered Species of Wild Fauna and Flora (CITES): Lessons Learned on Implementation and Compliance

**DOI:** 10.1007/s10991-020-09267-8

**Published:** 2020-10-07

**Authors:** Tanya Wyatt

**Affiliations:** Department of Social Sciences, Northumbria University, Newcastle, UK

**Keywords:** Wildlife trade, Wildlife law, Wildlife trafficking, CITES, Wildlife law enforcement

## Abstract

Unsustainable and illegal wildlife trade are contributing to the unprecedented levels of biodiversity loss and possible extinction of one million species. Law enforcement and the criminal justice system have a role to play in helping to regulate and monitor such trade. The main international instrument to regulate wildlife trade is the Convention on International Trade in Endangered Species of Wild Fauna and Flora (CITES). This mixed methods study researched the lessons learned and best practice in regards to implementation of and compliance with CITES. As part of the study, three countries were identified as case studies and Canada was selected as one of these. Lessons can also be learned from Canada’s Wild Animal and Plant Protection and Regulation of International and Interprovincial Trade Act, which is cumbersome to update when species protections change within CITES. Canada has several elements of good practice, such as the remit, effectiveness and relationships of the three CITES authorities located within Environment and Climate Change Canada, the public health approach to some wildlife imports, and the protection of native CITES species. CITES needs to be improved to further protect endangered species and lessons from Canada and other countries can contribute to this improvement.

## Introduction

One million species are facing human-caused extinction (IPBES 2019). After habitat loss, the second main threat to species is overexploitation, which includes unsustainable and illegal wildlife trade (IUCN 2016; WWF 2017; IPBES 2019). Overexploitation of wildlife is a ubiquitous problem that, as Schalow (2015) argues, the law deliberately and inadvertently avoids discussing. As animals and plants receive little protection in common law, international conventions are an important means to protect wildlife and habitat (Rodgers 2013). This article is a portion of the findings from a project that sought, in part, to contribute to remedying this lack of attention by gathering data on lessons learned in relation to the Convention on International Trade in Endangered Species of Wild Fauna and Flora (CITES). CITES is the key international legal instrument to combat both the overexploitation from the legal trade and the illegal side of the trade. Even though CITES has been adopted by 182 countries and the European Union, implementation of CITES legislation remains inconsistent, compliance at times lacking, and listed species still face extinction (CITES 2016; Reeve 2006; Wyatt 2013). Furthermore, the minimal research that there is related to CITES (Reeve 2002) has largely been limited to conservationists and political scientists with legal scholars having recently contributed to the discussions (see Wandesforde-Smith 2016 among others). There has never been an empirical study of the effectiveness of CITES (Reeve 2002) and there are a lack of empirical case studies related to implementation and compliance. A few policy or legal discussions about the pros and cons of CITES have been written (Aguilar 2013; Bowman 2013; Couzens 2013; Sands 2013; Scanlon 2013; Wandesforde-Smith 2016), but these are theoretical. The importance of an empirical study is the evidence/data collected can have practical implications for improving legislation and thus species protection and survival.

Without national legislation implementing CITES, the regulation of international wildlife trade would be impossible. As Oldfield (2003) has noted, lack of enforcement is often blamed when wildlife populations decline. Yet the fault may be with the design and implementation of the regulation (Oldfield 2003), but there has been very little exploration of national implementation (Wandesforde-Smith 2016) or of projects and approaches that might improve CITES (Bowman 2013). In addition, there are a range of, often poorly understood, factors which are likely to influence the apparent effectiveness of CITES, such as the soft law mechanisms integral to implementation and compliance (i.e. international action plans, reviews of significant trade, non-detriment findings and so forth) (Aguilar 2013; Munoz 2016). Another of the factors may be the communication and cooperation between national CITES Management and Scientific authorities, which will affect effective implementation even when the legislation strictly adheres to the Convention. It may be that the regulation regimes in countries with species decline and lack of compliance can benefit from improved regulation in the form of legislation, implementation and compliance mechanisms rather than or in addition to improved enforcement.

This article focuses on Canada’s implementation of and compliance with CITES. First, I will provide background on CITES, the relevant legislation in Canada and the structure of Canadian wildlife law enforcement. Then, I will briefly summarise the methods used to collect data, which led to Canada being one of three case studies. Lastly, supported by the relevant literature, I will share the lessons learned from Canada’s approach to implementation of and compliance with CITES. The aim is to potentially improve Canada’s practice, but also to demonstrate to the other parties to CITES possible changes to their own implementation and compliance approaches.

## Background

### The Convention on International Trade in Endangered Species of Wild Fauna and Flora (CITES)

CITES, or the Washington Convention, was drafted in 1973 and came into force in 1975. The main aim of CITES is to ensure that trade is sustainable and does not threaten the survival of species. As with all international conventions, membership of CITES is voluntary and parties that sign the convention agree to implement and enforce the convention as written. Since the beginning, the system in place has been three appendices of listed species that reflect the level of protection that the species is given. Additions and removals of species from these lists (as well as Resolutions and Decisions that address the mechanics of implementation and compliance and supplementary issues) are voted on every three years at the Conference of the Parties. Each party has one vote and can propose changes to the listing of species. At the time of writing, there are 669 fauna and 334 flora species listed in Appendix I, which means trade should only happen for scientific purposes (CITES No Date). There are an additional 4952 animals and 29,644 plants listed in Appendix II (CITES No Date) that are allowed to be traded within set quotas and with the proper export permits. The Scientific Authority in each country sets the quotas based upon population data, species reproduction and other relevant information. Sticking to the quotas is meant to ensure that trade will not affect species survival. Yet, in 2011 there were 625 Appendix I animals and 301 Appendix I plants (Wyatt 2013). For Appendix II listed species in 2011, there were 4685 animals and 29,105 plants. This indicates that hundreds of species are added to the CITES appendices (particularly Appendix II) at the Conference of the Parties. Given the number of species traded, the number of species listed in CITES because trade is posing a threat to their survival, and that the number listed keeps increasing, it is vital to improve CITES.

Implementation of the convention means that parties are required to transpose the convention into their national legislation. There are four elements to this transposition: there are designated Management and Scientific authorities; violations of CITES are prohibited; there are penalties in place, and confiscations of illegally traded and/or possessed wildlife and wildlife products are possible. The Management authorities oversee the permit system that accompanies and tracks trade transactions. The Scientific authority, as mentioned, establishes the quotas to be followed. There can be several Scientific authorities. For instance, in the UK both the Joint Nature Conservation Committee (JNCC) and the Royal Botanic Garden at Kew are Scientific authorities. Parties have the discretion to prohibit violations as they deem appropriate. That means prohibition does not have to be criminal and can be an administrative or civil offence. Using the UK as an example again, minor or Schedule 2 offences of the Control of Trade in Endangered Species Act 2018^1^ (which implements CITES) are issued compliance and/or stop notices. Schedule 1 offences, which prohibits trade in CITES species in violation of the convention, are criminal offences. In the UK context, penalties range from up to six months imprisonment or a fine to up to five years in prison or a fine or both depending upon the severity of the offence. Again, the penalty applied for CITES violations is not proscribed by the convention; there simply needs to be some sort of penalty. Finally, when illegal wildlife is uncovered during transit or in someone’s possession, Parties are required to have legislation to enable the confiscation of the wildlife. The UK legislation allows for forfeiture of wildlife as well as forfeiture of vehicles, equipment or other items used to commit the offence. This is more than required by CITES.

The CITES Secretariat categorises the national legislation of Parties into three groups (CITES 2016). The CITES National Legislation Project (NLP) is the initiative of the Convention started in 1992 that oversees these categorisations. The NLP is designed to analyse and assist members in drafting and implementing legislation that will meet CITES standards. Category 1 indicates the legislation is generally believed to meet the requirements of implementation. In 2018, ninety-two countries or just over 50% of countries were in this category. For countries in Category 2, their legislation is believed generally not to meet all of the requirements for implementation (46 countries/25.3%) and in Category 3 the legislation is believed generally not to meet the requirements for implementation (36 countries/ 19.8%; eight countries have recently joined and not been assessed). In addition, the CITES Secretariat has produced a Model Law and checklist for implementation, but these are general guidelines. The Standing Committee of CITES is where the business of the Convention takes place, including scrutiny on compliance, which may involve sanctions if countries do not meet the reporting requirements and/or make a concerted effort to implement the Convention (CITES 1992). Now that the general structure of CITES is clear, I will detail Canada’s specific approach to CITES.

### Canada

Canada ratified CITES a few months before it came into force in 1975, so it is one of the original Parties. According to Reeve (2002), a technical mission to Canada in the late 1990s was complimentary of Canada’s wildlife law enforcement effort. At the time, there was only 35 officers tasked with protecting and monitoring wildlife in one of the largest countries in the world (Reeve 2002). There was evidence of “close cooperation with customs, training, innovation at ports such as digital cameras for identification” and excellent identification manuals (Reeve 2002: 208). In terms of CITES’ National Legislation Project (NLP), Canada is a Category 1 country. This means the four main criteria outlined in the previous section are all present. Canada also has an Enforcement Authority with full peace officer powers of investigation and arrest though this is not required by the Convention. All three authorities are part of Environment and Climate Change Canada and share the same headquarters. In Canada, CITES is implemented through the Wild Animal and Plant Protection and Regulation of International and Interprovincial Trade Act (1996) (WAPPRIITA), an environmental law specifically to implement CITES. Acquiring of wildlife must be legal by the regulations from the place where the wildlife was taken, including other countries and each of the Canadian provinces (see more about law enforcement and regulation below). This makes WAPPRIITA somewhat unique by taking into account multiple jurisdictions laws and harmonises the legislation of Canada and the United States, where the US Lacey Act (2008) also makes trade unlawful if it violates individual State or foreign laws.

In regards to penalties, fines range from CAD 5000 to 12 million and imprisonment can range from six months to five years. When issuing fines, financial hardship is taken into account. Corporations are given the highest fines and the court can mandate that their shareholders are made aware of the nature of the offence and of the punishment given. Confiscations of illegally trade and/or possessed (the fourth element of implementation) are covered in WAPPRIITA, although there is no specific provisions for wildlife, who are alive. The money from fines for violation of WAPPRIITA/CITES may go into the Environmental Damage Fund to support conservation and restoration. The court may also order support for conservation, research, and/or education activities and a three-year follow up with the offender.

**Fig. 1:**
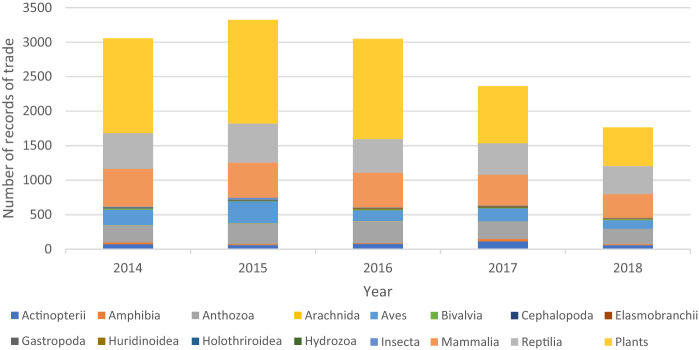
CITES species imported to Canada 2014 to 2018 (UNEP-WCWC 2020)

Of the several thousand CITES-listed species, Canada has relatively few native species on those lists. In terms of exports of CITES species then, oversight is only needed for 13 animal species (seven mammals mostly cetaceans; five birds; one reptile—the leatherback turtle) and nine plant species (six orchids, the Eastern prickly pear cactus, American ginseng and golden seal). Import of CITES species makes up most of the CITES trade for Canada (see Fig. 1). Between 2014 and 2018, most CITES imports to Canada were of plant species, mainly orchids. The other notable classes of imports were Reptilia, Mammalia, and Aves (birds). There was some trade in corals (Anthozoa) as well. Overall, imports appear to be for the pet industry (live reptiles, mammals and birds and coral for aquariums), and the luxury fashion (reptile skins) and horticulture (orchids) markets.

### Canadian Wildlife Regulation and Enforcement

As mentioned, CITES requires Parties to have a Management and a Scientific Authority. Both of these authorities in Canada are located within the Canadian Wildlife Service, which is part of Environment and Climate Change Canada. Canada’s Enforcement Authority—the Wildlife Enforcement Directorate (WED)—is also a part of Environment and Climate Change Canada. As stated, all three are located in the same headquarters, which is located in Ottawa. The Management Authority oversees the allocation of CITES export and import permits. The Scientific Authority is tasked with obtaining all of the scientific evidence underpinning quotas and listing recommendations. The WED enforces federal wildlife legislation throughout Canada. Under the Canadian Department of Environment Act (1985), the Minister of Environment and Climate Change has the power, duty, and function to preserve and enhance the quality of the natural environment (including water, air and soil). This Ministry also coordinates the relevant policies and programmes of the Canadian government related to renewable resources, migratory birds, and other non-domestic fauna and flora (Environment and Climate Change Canada 2019).

Thus, while the Canadian Wildlife Service and the WED ensure compliance and enforcement of CITES, these agencies also oversee the compliance and enforcement of other wildlife legislation. This includes the Species at Risk Act (2002), the Migratory Birds Convention Act (1994), and the Canada Wildlife Act (1985), in addition to the Wild Animal and Plant Protection and Regulation of International and Interprovincial Trade Act (1996) (WAPPRIITA). The enforcement of this legislation.

has evolved significantly over the last century. Long-time the responsibility of the Royal Canadian Mounted Police, in the 1970s and 80s the mandate moved to Environment and Climate Change Canada (ECCC), where it has resided first in the Canadian Wildlife Service and, since 2006, in the Enforcement Branch (WED 2019: I).

Furthermore,

The role of enforcement has changed as well. Our enforcement officers are now responsible for protecting over 400 species of migratory birds including nests and habitats; 146 protected areas, international and interprovincial trade in wildlife for over 36,000 species; and most recently over 560 species at risk in areas of federal jurisdiction (WED 2019: I).

It is important to note, and will be discussed in more detail later after a discussion of the methods, that the three authorities’ focus is on wildlife, rather than most jurisdictions where wildlife is one of many portfolios being handled by the authorities and law enforcement.

## Methods

The research underpinning this article was a two-year qualitative mixed methods research project that started on 1 May 2018. First, a content analysis of the legislation of the 183 Parties was conducted. For 112 Parties, CITES legislation or review of the legislation is available in English. Twenty countries’ legislation is in Spanish and Russian, which I have proficiency in. Google translate was therefore used for 47 Parties. Four Parties’ legislation could not be read as the text could not be translated or was unclear (three in Arabic; one in Somali). Second, a Delphi iterative survey unpacking the analyses of the legislative content analysis was launched. There were 32 participants in the first round (14 women and 16 men; 17 government representatives, 4 from civil society, 8 from academia, and 3 other; 13 participants were based in Europe; 7 in North America; 6 in Africa; 3 in Oceania/Pacific and 1 each in Asia, Central/South America, and no answer)and 21 in the second (7 women; 13 men; 1 prefer not to say; 17 government representatives, 2 from academia, 2 other; 5 participants were based in Europe, and 4 each in Africa, Asia, Central/South America, and North America). Delphi iterative surveys are targeted at experts, who in later rounds comment on the anonymous responses from the previous round in order to test new ideas and try to achieve consensus on solutions proposed. One of the main outcomes of the survey was the identification of three case studies of best practice and/or lessons learned. These are Canada, Indonesia and South Africa. Following the case study selection, in-depth interviews were conducted with 20 interviewees and lasted between 30 and 65 min each. Interviews further developed the best practice and lessons learned of the three case study countries, including Canada (the other case studies are the focus of other articles). Interviewees are kept anonymous and confidential. Full ethical approval was granted before any data was collected.

## Findings

As one survey respondent commented, “The Canadian and US models are quite good, although neither is perfect”. That is to be expected considering, as several interviewees remarked, that CITES can be complicated in terms of transposing the Convention into national legislation and then implementing and complying with the main text of CITES as well as dozens of supporting Resolutions and Decisions mentioned above (though these are not mandatory). In the case of Canada, there were three main lessons for other countries, which remain areas for Canada to improve—involvement of all core agencies, priority of some species over others, and updating legislation to reflect newly protected species. Overall, several elements of Canada’s implementation and compliance could be considered as best practice, in particular, the specialisation of their CITES team, the inclusion of public health measures, and the protection of their native species, which includes consultation with local and indigenous people.

### Lessons Learned

#### Involvement of Core Agencies

**Fig. 2:**
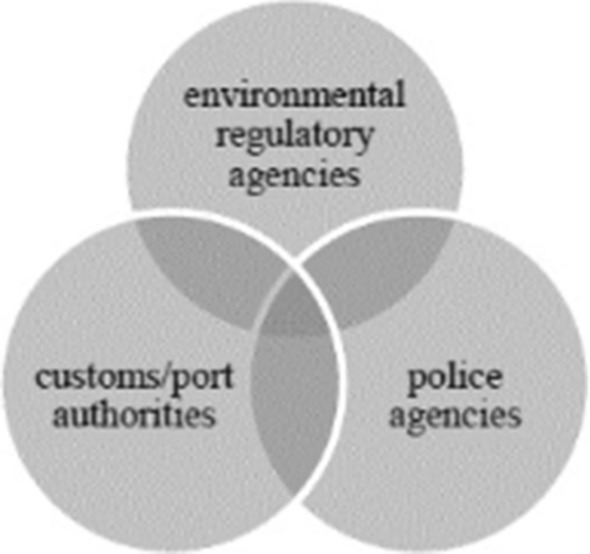
The core agencies involved in combatting transnational organised crime (Pink 2013a: 3)

One ongoing challenge in regards to CITES in Canada is the lack of the border agency’s full engagement with CITES (personal communication, February 2020). Pink (2013a) proposes there are three core agencies needed to combat transnational environmental crime, like wildlife trafficking. As seen in Fig. 2, these are police agencies, environmental regulatory agencies and customs/port authorities. In the case of Canada, the WED are the police agency and the Canadian Wildlife Service (the CITES Management and Scientific Authorities) are the regulatory agencies. “The single most important requirement for effective environmental law enforcement is co-operation between regulatory agencies and traditional law enforcement officials” (Situ and Emmons 2000: 140). Thus to be the most effective, it is important that police, regulators, and customs work together. This seems particularly pertinent in the context of CITES, where checking compliance and enforcing the legislation is critical at borders—a key point during trade and trafficking.

As with possibly every border agency in the world, it appears in Canada that wildlife and CITES are not their priority. Interviewees stated the problem is the prioritisation of drugs, terrorism and contraband items, such as cigarettes and so forth, means that illegal wildlife are not actively being searched for at ports and borders. The scale and scope of illegal wildlife trade in CITES species then can only be estimated as there is an unknown amount of smuggling taking place. This unknown figure may be particularly high for illegal wildlife trade, since it is not a priority (van Uhm 2016; Wyatt 2013). Further efforts to involve customs and border agencies in Canada and elsewhere are important to continue to improve compliance and enforcement. It is widely recognised that effective environmental, or in this case specifically wildlife, law enforcement is impossible without a multilateral response, including cooperation and coordination between agencies at the national, regional and global levels (Clifford and Edwards 2012; Horne 2013a, b; Pink 2013a).

#### Priority of Some Species

Within wildlife trade, there is evidence of priority being given to some species over others (Sollund 2019; Wyatt 2013). This priority, or speciesism,^2^ seems to usually be grounded in perceived charisma of the wildlife. Non-human animals like elephants, rhinoceros and tigers have been the focus of attention in the media and at CITES. Reptiles, amphibians, and fish (and plants) are less likely to be the focus of campaigns presumably because of speciesism. In the context of Canada, Vanderzwaag and Hutchings (2005: 220) state, “The management of risk to marine wildlife species brings to the scholarly forefront deep societal differences in values and interests and varying views on how precautionary decisions should be.” Thus, marine species and their trade may not be as scrutinised as terrestrial species and their trade. This was supported by one interviewee, who thought the National Legislation Project categorisations were only judged on implementation of CITES legislation for terrestrial species and disregarded whether or not provisions have been put in place for marines species. Linked to the apparent lesser regard for the plight of marine species in Canada, is the unclear relationship between Canada’s Species at Risk Act (2002) (SARA) and WAPPRIITA (Vanderzwaag and Hutchings 2005). SARA is meant to determine how to protect native species. Under SARA, fishing licences can be issued for threatened fish species. Whereas this is not relevant to CITES because no Canadian native fish species are listed in CITES appendices, the significance is that fish can be exploited in Canada under SARA even when they are a protected species. This indicates that not all species are protected to the same degree even when afforded the same protection status.

#### Updating Legislation to Reflect Newly Protected Species

The main area for improvement in regards to Canada’s implementation and compliance with CITES is related to WAPPRIITA. Several interviewees pointed to Canada’s problematic approach to adding new species to the CITES appendices. As mentioned, at each Conference of the Parties every three years, dozens of species are added to the CITES lists. In the Canadian context, these additions must be approved through public consultation and Parliament after the close of the Conference of the Parties. While public consultation is normally a positive measure, as will be discussed shortly, in this context it creates hurdles for timely implementation and compliance. Under CITES, new listings of species should come into force 90 days after the close of the Conference of the Parties. With such a lengthy process for adding endangered and threatened species to WAPPRIITA, Canada always has to enter reservations^3^ because they are unable to list the new species in the timeframe required by CITES. Wildlife that should be protected and traded with permits and so forth are not afforded these provisions in a timely manner in Canada. Although further exploration is needed, there are two main concerns with this. First, this is a potential loophole, which could be exploited by using Canada as a location to stockpile wildlife that will become listed, but has not yet in that jurisdiction. Second, depending on the time length of the reservation, trade in a species could continue at volumes endangering their survival and bring them even closer to extinction before the protection measures are implemented. As Pink (2013b: 9) notes, tensions and disconnects “can arise when on-ground application of legislation is not synchronised with the existing legal and policy context”.

### Best Practice

Apart from these lessons, Canada appears to have several elements of good practice that might help other Parties to improve: a specialised set of authorities, an element of public health integration, and their approach to protecting native species, including local and indigenous communities’ consultations, which has been identified as critical to protecting wildlife (see Roe and Booker 2018 among others for a complete discussion of community engagement in conservation).

#### Specialised Authorities

For Horne (2013a, b) the optimal wildlife crime policy response must be (1) proactive and intelligence based, (2) multifaceted, addressing many aspects of the problem, (3) multilateral, involving cooperation between several actors, and (4) monitored, evaluated, and adapted as necessary. There is evidence of each of Horne’s four criteria in the Canadian context, particularly as interviewees highlighted, due to the strengths of Canada’s authorities. As a foundation, Canada’s approach is effective because they have defined roles between three distinct authorities. Furthermore, each of the authorities is dedicated to and specialises in wildlife, and “Special purpose law enforcement agencies are those that perform some enumerated duty in specified jurisdictions that are beyond the scope of traditional law enforcement agencies” (Patten et al 2015: 751). In the Canadian context, the ‘law enforcement’ is broad to encompass the regulators as well.

Specialisation enables agencies to be proactive and intelligence based. This links to the fact that law enforcement of transnational environmental crime (such as wildlife trafficking) requires a unique skill set (Pink 2013b). These staff need to be experts in the relevant investigative techniques as well as have working knowledge across the administrative, civil, and criminal laws and sanctions that govern the environment and wildlife (Pink 2013b). This is in addition to the scientific and environmental knowledge that is needed. “Mainstream law enforcement agencies, whilst appreciating the (investigative) enforcement aspects, tend not to have a full understanding of the environmental or scientific aspects of TEC [transnational environmental crime]” (Pink 2013b: 21). These scientific and environmental skills are necessary to be proactive and to gather intelligence.

Much of the evidence for the effectiveness and benefits of specialisation comes from environmental courts. For instance,

…it has been demonstrated empirically, that when specialist environmental courts are in place (or there are trained judicial officers with specialist environmental knowledge within generalist systems), there is greater likelihood of both prosecution of offenders and greater use of appropriate sanctions (White 2013: 269).

Environmental specialisation in courts has also been found to likely reduce costs for handling human and financially resource intensive cases (Pring and Pring 2009). Furthermore, “Specialisation enables use of special knowledge and expertise in both the process and the substance of resolution of these problems” (Preston 2011: 29).

The specialisation of the Canadian authorities in addition to supporting being proactive and intelligence based allows for multifaceted approaches to the problem. Wildlife crime and trafficking, as mentioned, requires not only investigation experience, but also additional knowledge of species and, in Canada, understanding of the provincial, national and international laws. Furthermore, the regulators overseeing the legal trade also need the knowledge of species and the provincial, national and international laws governing those species’ trade. The different layers and diverse knowledges lends to possible prevention and disruption strategies on numerous fronts. One multifaceted approach that is another strength of Canada is their engagement with wildlife law enforcement on the global level. For example, key figures in Canadian law enforcement, such as the Director General of the Wildlife Enforcement Directorate, Sheldon Jordan, have been core members of international efforts to tackle wildlife crime and trafficking. In this case, Director Jordan served as the Chair of INTERPOL’s Wildlife Crime Working Group. INTERPOL is a member of the International Consortium on Combatting Wildlife Crime (ICCWC) along with CITES, the World Customs Organisation, the World Bank, and the UN Office on Drugs and Crime. Together the five supranational organisations develop and deliver trainings, guidelines and toolkits to support global initiatives to combat wildlife crime and trafficking. ICCWC has also supported global law enforcement operations to disrupt illegal trade in the last few years. Canada is an active participant in these multi-lateral law enforcement operations. So, while Canada does not provide large amounts of funding to CITES (i.e. the EU) or global programmes to combat illegal wildlife trade (i.e. the UK and US), they capitalise on their strengths by supporting international activity. Pink (2013b) proposes international exposure ensures staff can see the bigger picture and thus the trends, which makes them more effective.

In regards to Horne’s third criteria—multilateral cooperation between actors—as detailed in the Background, Canada, for the most part, does this from the outset by having a structure to ensure compliance with CITES that involves three agencies that are both regulators (the Management and Scientific Authorities in the Canadian Wildlife Service) and enforcers (the WED). And as just mentioned, the multilateral efforts of Canada’s CITES team extends to the international level. In relation to the fourth criteria for monitoring, evaluating and adapting, the adaption appears evident in the re-structuring of wildlife law enforcement in Canada, also detailed in the Background section. Once part of the federal traditional agency, the Royal Canadian Mounted Police, wildlife law enforcement was separated out into a specialised unit in the mid-2000s. The fact that the three authorities are co-located within the same building was thought by one interviewee to be an element of their effective working relationship. This may be linked to the organic monitoring and evaluating of each other that takes place when working in close proximity. The co-location may also lend to collegial and tight working relationships. The effective working relationship of the three authorities extends beyond Environment and Climate Change Canada. Interviewees remarked that Canadian authorities also have good working relationships with international partners (particularly the US Fish and Wildlife Service) and the Canadian authorities of other conventions (i.e. Convention on Biological Diversity and so forth). These also speak to Horne’s criteria two and three of an optimal wildlife crime policy as collaboration with domestic and international partners enhances both the multifaceted approach to the problem and multilateral cooperation.

#### Public Health Integration

One interviewee indicated that better cooperation could take place between Health Canada and Environment and Climate Change Canada. However, this raises an interesting and, in the light of the coronavirus pandemic, potentially useful element to Canada’s approach to wildlife trade.^4^ Particular species who are known to be pathogen carriers that can transmit to other animals and to humans are not allowed to be imported into Canada. Other species who are a high risk of transmitting disease and so forth are required to have additional documentation—health permits—evidencing the animal’s health. Thus, there is a restriction on the import of turtles, tortoises, and their eggs because they risk spreading serious diseases, in particular Salmonella. For pet turtles and tortoises, they all need an import permit and must have been in the owner’s personal possession in the country where the animal was taken from and must be accompanied by the owner (CFIA 2020). New species of frogs must be risk assessed to determine their likelihood of carrying and transmitting diseases (CFIA 2020).

Trade of other species impacted by this public health approach are pet birds, which cannot be imported from Bangladesh, China, Egypt, India, Indonesia, or Vietnam. This is due to the endemic nature of avian influenza in these countries, which is highly contagious (CFIA 2020). Primates can only be imported to Canada if for a zoo, show, exhibition, or research use. All primate imports require a permit from the Canada Food Inspection Agency (CFIA 2020). Pet fish do not require a CFIA issued import permit if there is proof of ownership and a declaration that the owner will be keeping the pet fish in a home aquarium (CFIA 2020).

Marine species, excluding pet fish, seem to be the subject of a majority of the import permits to Canada. Likely, this stems from the commercial nature of the trade and that marine species are entering the food chain. An Aquatic Animal Health Import Permit is needed for species who are susceptible to a range of diseases (CFIA 2020). There are approximately 115 species of crustacean, 56 molluscs including many abalone species, and 146 Finfish (like salmon and trout, but also white sturgeon and blue fin tuna), which require permits (CFIA 2020). According to the Canada Food Inspection Agency website, “import permits contain specific requirements based on the disease risks associated with the aquatic animal, the origin, the commodity type, the end use of the aquatic animals in Canada and other relevant health information”. While not all of these marine species are listed in the CITES appendices, there a few which these regulations might affect. More importantly, integrating public health into wildlife trade regulation is likely to be an essential element moving forward to combat future pandemics.

#### Protecting Native Species

As outlined above, WAPPRIITA not only criminalises taking or possessing of wildlife that is illegal in another country, it also criminalises the illegal taking or possessing of wildlife between Canadian provinces. This added layer of protection for native species and scrutiny of legality at the provincial and international level appears to contribute to Canada’s effective implementation of CITES. Several respondents to the second round of the Delphi iterative survey felt that strong protection of a country’s native species constituted best practice. Admittedly, as one interviewee pointed out, Canada has fewer species to monitor than other CITES countries. However, their approach may still be adaptable to other contexts.

Furthermore, according to interviewees, Canada has made a concerted effort to include the voices of indigenous communities in wildlife management decisions, particularly in regards to the polar bear. This does come after years of poor communication with Canada’s First Nations (personal communication, February 2020). Several interviewees felt that the relationship with the First Nations was now a good one, where indigenous communities were active participants in crafting and implementing the regulations. Numerous Delphi survey respondents and interviewees expressed that inclusion of local and indigenous communities in designing, implementing and enforcing wildlife regulation is a critical element of any CITES implementation (though not required) if wildlife are to be protected from overexploitation and illegal activity.

## Discussion and Conclusion

The threat of extinction and the loss of species to overexploitation and illegal trade demands urgent attention. CITES is the main global instrument for addressing overexploitation and illegal trade in the international context. While it has garnered support and praise during its 45 years of existence, there is work to be done to make it better. Canada has several lessons to teach other countries and several practices that all could lead to the improvement of CITES implementation and compliance. In terms of lessons, it is important that all agencies that have even a small role in wildlife trade are integrated into putting CITES regulations into practice in the national context. In the case of Canada, while the Management, Scientific and Enforcement Authorities seem to be effective and well connected, the border/customs officers and the Canada Food Inspection Agency appear to need further inclusion. Another lesson is that listed species should be given the same protection. Marine species need to be inspected, looked out for, and investigated as any other species. Lastly in terms of lessons, legislation is most efficient and effective if written in such a way that the CITES’ lists as a whole are recognised and thus updated at the national level automatically. Otherwise, as in the case of Canada, protection of endangered species is delayed.

The practices of Canada that other countries should consider adapting to their own contexts are: first, authorities are likely at their best when they are wildlife/CITES specialists. Furthermore, it is important to have distinct roles and good working relationships between the authorities. Both specialising and clarity of roles enable proactive enforcement that can be intelligence based. Specialisation also supports multifaceted approaches to problem solving that in terms of wildlife crime and trafficking can involve traditional law enforcement skills of investigation and so forth, but also entail the critical knowledge around the environment and wildlife. In addition to multifaceted approaches, as Horne (2013a, b) suggests optimal wildlife crime policy is also multilateral. This seems to be a particular strength in Canada where there is an effective and close relationship with the three CITES authorities. The multilateral strength of Canada’s approach extends beyond the three authorities to working with other Canadian agencies, the US, and international partners. The cooperation and collaboration demonstrated in these working relationships are worth trying to replicate.

The other best practices of Canada are the approaches to the integration of public health and the protection of native species. Prior to the coronavirus pandemic, the added health permit for some animals simply added a layer of documentation that could be used for further scrutiny during trade. Now with the current pandemic continuing to infect more people and take more lives, health permits may become much more prevalent in global wildlife trade. The exact mechanisms for the implementation of these permits by Canada are worth further research to inform the post-pandemic regulation of wildlife trade. The approach to protecting native wildlife was also thought to be best practice. This stems from the national measures put into place to protect Canadian wildlife, but particularly stems consulting local and indigenous communities’ in creating these national measures as well as weighing in on international proposals.

Overexploitation and illegal wildlife trade are global problems, so being actively involved in international activities be that through CITES, INTERPOL or law enforcement operations can only help to combat these problems. Here, too, Canada sets a good example of engaging with the various fora that are available to try to tackle wildlife crime and trafficking. CITES implementation and compliance needs to improve on numerous fronts to help stop the decline of wildlife, but there are lessons to be learned and best practice to emulate from Canada and other CITES member countries in order to do so.

## Data Availability

Data sets can be found here: https://drtwyatt.weebly.com/cites-implementation-and-compliance.html.

## Footnotes

1 UK legislation at the time of writing also transposed European Union Directives. In the case of CITES, these are Council Directive 92/43/EEC of 21 May 1992 on the conservation of natural habitats and of wild fauna and flora; Council Directive 2008/99/EC on the protection of the environment through criminal law; and COUNCIL REGULATION (EC) No 338/97 on the protection of species of wild fauna and flora by regulating trade therein. So the EU requires criminal sanctions for CITES, but CITES does not proscribe this.

2 Speciesism is the discriminatory treatment and lack of consideration for non-humans (Ryder 1970).

3 A reservation is a statement made in order not to be bound by CITES provisions concerning a particular species listed in the Appendices.

4 Canada is not the only country to have an additional layer for trade that relates to screening for diseases and pathogens. Germany, Hungary, Poland and Serbia appear to have similar requirements.
